# Long Term Follow-Up of a Successful Lower Limb Replantation in a 3-Year-Old Child

**DOI:** 10.1155/2015/425376

**Published:** 2015-04-02

**Authors:** Akbar Jaleel Zubairi, Pervaiz Mahmood Hashmi

**Affiliations:** Aga Khan University, Karachi 74800, Pakistan

## Abstract

Replantation of the lower extremity has controversial indications but nevertheless it may be considered in carefully selected patients who present early and are expected to show good functional recoveries. Here we present a successful replantation in a 3-year-old boy who has made excellent recovery with no functional deficit evident at 12 years of follow-up. He sustained a traumatic amputation at the level of distal tibia when he fell of a “Qing Qi” (motorcycle rickshaw). Replantation was attempted at 8 hours cold ischemia time with the tibia shortened 4 cm and all tendons, vessels, and nerves repaired. Patient required a second procedure during the same hospital stay for skin coverage. Patient made good recovery with ambulation without support at 6 months, less than 3 cm limb length discrepancy, plantar and dorsiflexion power 4/5, and recovery of sensation over the foot. Now at 12 years of follow-up patient has a normal gait and has integrated into society with no functional deficit. Considering the functional outcome of our case, replantation should be attempted whenever possible and feasible especially in children.

## 1. Introduction

Replantation of the lower extremity has controversial indications because crushing and avulsion of the involved parts make the procedure difficult to perform and the results of modern prostheses are better than a poorly functional replanted limb [1, 2]. Nevertheless replantation may be considered in carefully selected patients who present early and are expected to show good functional recoveries [3–6].

Here we present a successful replantation in a 3-year-old boy who has made excellent recovery with no functional deficit evident at 12 years of follow-up.

## 2. Case Description

**Figure 1:**
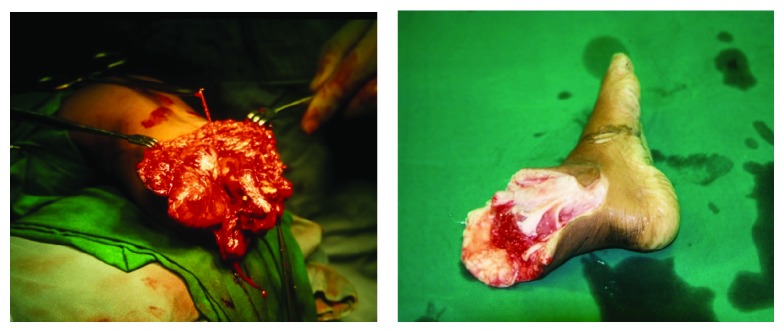
Preoperative.

A 3-year-old boy presented to our emergency department six hours after sustaining a traumatic amputation of his left lower limb when he fell of a “Qing Qi” (motorcycle rickshaw). The patient had received first aid from a local hospital and then was referred to our institute for hope of replantation. At presentation he had a pulse rate of 160/min and blood pressure of 108/64 mm Hg. The left leg was severed 10 cm distal to the knee joint and the amputated foot was being carried in a polythene bag filled with ice (Figure 1). There were no other systemic or limb injuries. Decision to attempt replantation was taken considering the age of the patient and borderline ischemia time even though the mechanism of injury was not in favor of this decision.

**Figure 2:**
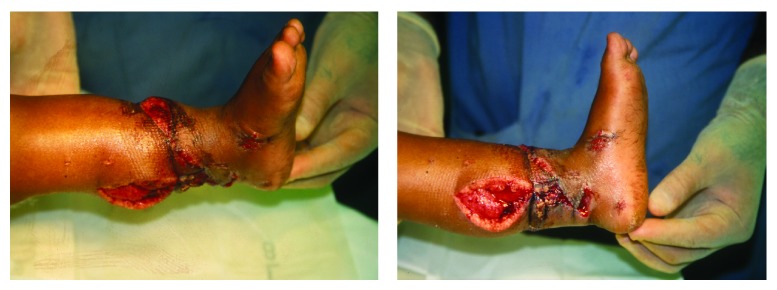
Postoperative.

Replantation was started at 7 hours cold ischemia time after adequately washing both the stump and the amputated foot. The tibia was shortened 4 cm to facilitate tendon, nerve, and soft tissue approximation and stabilized with a four-hole 3.5′ dynamic compression plate with 2 proximal and 2 distal screws. Coaptation of the tendons to provide stability to foot was undertaken in the following sequence: tibialis anterior, tendo-achilles, tibialis posterior, flexor hallucis longus, extensor hallucis longus, and the toe extensors. Primary anastomosis of the anterior tibial artery along with 2 accompanying veins was done. Defect in the posterior tibial artery was bridged with a reverse saphenous graft harvested from the opposite leg and the ipsilateral great saphenous vein was primarily anastomosed end to end. The tibial and peroneal nerves were primarily repaired and the skin was loosely tagged to provide temporary cover to the plate and anastomoses site (Figure 2). Immediate postoperative period went uneventful with regular dressing changes. Skin grafting for the skin defects over the medial (3 × 7 cm) and lateral (2 × 4 cm) sides of the wound was undertaken after 10 days during the same hospital stay (Figure 2).

**Figure 3:**
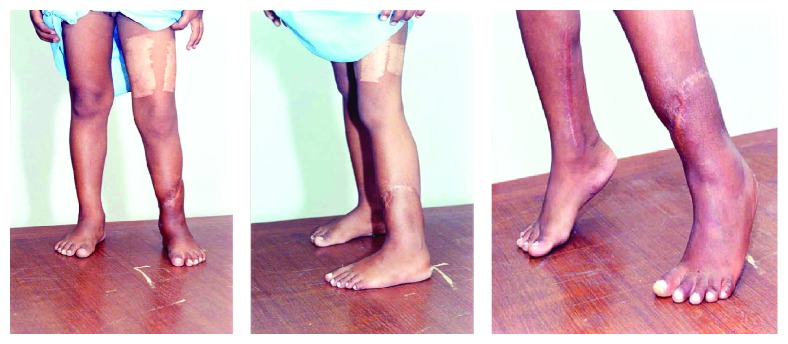
6-month follow-up.

Subsequently the patient made good recovery with recovery of skin sensation over the foot at 6 months, with less than 3 cm of limb length discrepancy, good plantar, dorsiflexion power of 4/5 on the BMC scale, and ability to ambulate without support (Figure 3). The plate was removed at 2 years and tenolysis of the long toe extensors was done in the same setting.

**Figure 4:**
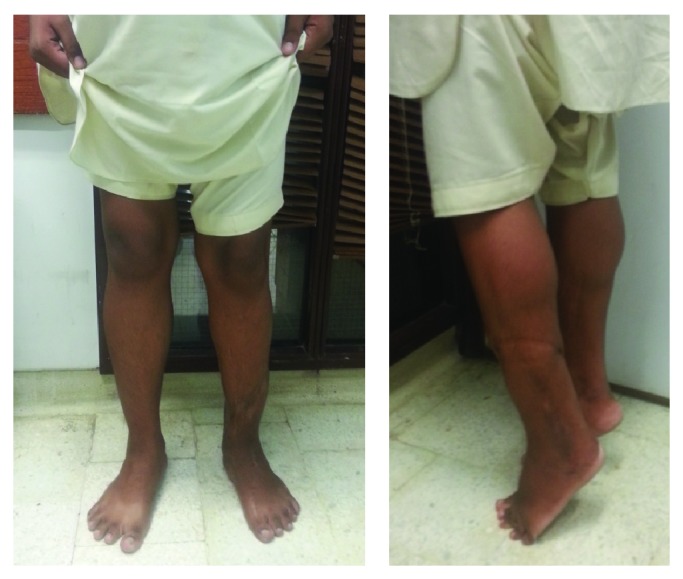
12-year follow-up.

Now after 12 years of follow-up the patient has a normal gait with no limp. He does not require a shoe raise for his negligible limb length discrepancy. He has 5/5 power of plantar and dorsiflexion with ability to walk on his toes and heels, respectively (Figure 4). He has integrated back into society with no functional deficit or handicap.

## 3. Discussion

Replantation is a complex surgical procedure performed by microsurgeons requiring specialized intraoperative instrumentation and postoperative care. Even though reports of replantations are reported in the literature since the 1960s there are still no reports of replantations from our country. In our resource constrained country availability of a microsurgeon and surgical instrumentation is scarce. As a result most children and adults alike end up in an amputation with no choice of salvage available. Rarely do such patients arrive in time to a facility with available resources where such an attempt can be made.

In the developing countries where patients finance themselves the debate between a nonfunctional replanted limb versus a functional prosthesis has a different angle to it. Attempting to salvage the extremity with the requirement of multiple procedures may be expensive but even good functional prostheses are scarce, expensive, and not within the reach of the common man [7, 8]. Similarly lack of proper facilities for the physically impaired at public places hinders their smooth integration into society [9]. These factors result in patients faring better with a less than optimal functional limb as compared to a prosthesis.

In children stump revisions, angular deformities and frequent change of prosthesis during growth spurts present another challenge with added cost [10–12]. The psychological impact of this disability to the children and their parents is also immense [13]. On the other hand replantation outcomes in children have been seen to be superior to adults with less wound complications and limb shortening [14, 15].

Children have a physiologically better healing response than adults with good bone healing secondary to a rich periosteal blood supply, faster soft tissue healing, less scar formation, improved nerve regeneration, easier joint mobilization, and enhanced tendon gliding [16]. Even though outcome of more proximal replantations may not be as favorable as the more distal ones many microvascular surgeons now feel that an attempt for replantation should be made in children due to their improved subsequent function and psychosocial adaptability [16].

Considering the functional outcome of our case we feel that replantation should be attempted whenever possible and feasible even in our society especially in children.
